# Spontaneous fractures of the mandible concept & treatment strategy

**DOI:** 10.4317/medoral.20716

**Published:** 2015-12-04

**Authors:** Anja Carlsen, Mette Marcussen

**Affiliations:** 1 DDS. Resident in oral and maxillofacial surgery. The Department of Oral and Maxillofacial Surgery, Aalborg University Hospital; 2 DDS. PhD. Student & surgeon in oral and maxillofacial surgery. The Department of Clinical Medicine, Aalborg University Hospital

## Abstract

**Background:**

Spontaneous fractures of the mandible dispose a surgical challenge in comparisons to fractures caused by trauma due to several complicating factors. Additionally: controversies exist concerning the terminology of the field.

**Material and Methods:**

We conducted a retrospective study of all patients with mandibular fractures, with exclusion of fractures of the coronoid process and the alveolar process, treated at the Department of Oral and Maxillofacial Surgery, Aalborg University Hospital, Denmark between February 2003 and February 2013. Data collected from the medical records included sex, age, cause of fracture, site of fracture, and treatment.

**Results:**

We identified 517 patients with 684 mandible fractures. Twenty-five of these were spontaneous fractures and 659 fractures were of traumatic origin. Condylar fractures rarely occur spontaneously, but constitute the majority of the traumatic fractures. Excluding these fractures from the analysis, we found a non-surgical approach in 14 of 24 (58%) of the spontaneous fractures and 110 of 376 (29%) of the traumatic fractures. This was statistically significant.

**Conclusions:**

We found a statistical significant difference in favor of non-surgical approach in spontaneous fractures and we discussed the treatment challenges of these fractures. We addressed the terminological controversies regarding pathological fractures, and suggested the term spontaneous fractures denoting a fracture occurring during normal jaw function being either pathological or non-pathological.

**Key words:**Mandibular fractures, spontaneous fractures, pathological fractures, traumatic fractures, treatment.

## Introduction

Controversy exists regarding the definition of pathological fractures. As stated by Coletti (1) some authors refer to “inadequate or minimal trauma causing a fracture trough a preexisting pathological bone lesion”. Other authors have contended the difficulty to define inadequate or minimal trauma and thus suggests the definition of a pathological fracture as “a fracture occurring through a preexisting lesion or in a diseased part of bone” (2). However, fractures of atrophic mandibles or iatrogenic fractures may fall into the group of pathological fractures even though no bone pathology is present, apart from weakening of the bone due to surgical trauma or atrophy (1,3,4). Therefore, neither definition covers the entire spectrum. In the present study, we apply the term spontaneous pathological fractures and spontaneous non-pathological fractures as fractures occurring during normal jaw function.

In the mandible, the spontaneous pathological fractures may occur in lesions of osteoradionecrosis, osteomyelitis and ARONJ (anti-resorbtive osteonecrosis of the jaw; former bisphosphonate-related osteonecrosis) or be caused by cysts, malignant or benign tumors. The spontaneous non-pathologic fractures may occur in mandibles weakened by atrophy or surgery, typically mandibular third molar surgery or bone harvesting procedures (1-14).

Owing to the very different healing potential of spontaneous pathological and spontaneous non-pathological fractures, the treatment strategy differs and depends upon local factors such as the nature of the bone pathology, options for buttressing, but also upon the patient´s general health including co-morbid diseases (1,3,4).

Contrary, in fractures due to trauma, the fracture pattern is generally straightforward allowing adequate buttressing and good bone apposition following reduction of the mandibular fracture (1). Moreover, the patients are usually younger and in a good health (15).

In this study, the difference in treatment strategy in terms of surgical versus non-surgical approach between spontaneous mandibular fractures and traumatic mandibular fractures is assessed and the challenges in handling spontaneous fractures is discussed.

## Material and Methods

We conducted a retrospective review of medical records and radiographs in patients with mandibular fractures treated at the Department of Oral and Maxillofacial Surgery, University Hospital in Aalborg, Denmark from February 2003 to February 2013. The study obtained institutional review board approval (The Data Protection Agency of the North Denmark Region), and informed consent was renounced.

All mandibular fractures were included with the exception of fractures of the coronoid process and the alveolar bone. These two types of fractures are quite abundant among the non-spontaneous fractures, but have never been reported among spontaneous fractures. Therefore, in order to secure comparability and avoid bias in the group of the spontaneous fractures, fractures of the coronoid process and the alveolar bone were excluded. Data collected included sex, age, cause of fracture, site of fracture, and treatment.

- Statistical analysis

Stata version 11.2 (StataCorp LP; College Station, TX) was used for statistical analysis. Each fracture was registered as one entity, thus a patient occurred two or more times if more than one fracture was present. Consequently, we introduced a cluster variable. Throughout, a *p*-value <0.05 was considered statistically significant. A linear model was set up, and we used a linear regression analysis to analyze statistical significant difference in treatment approach, sex and age between patients with spontaneous fractures versus trauma related fractures.

## Results

Through a 10-year period, we identified 684 mandibular fractures in 517 patients ( Table 1). Of these, 25 patients presented with 25 spontaneous fractures: 17 pathological and 8 non-pathological fractures; 14 (56%) patients were male and 11 (44%) were female with an average age of 60.6 years (range 16-90).

**Table 1:**
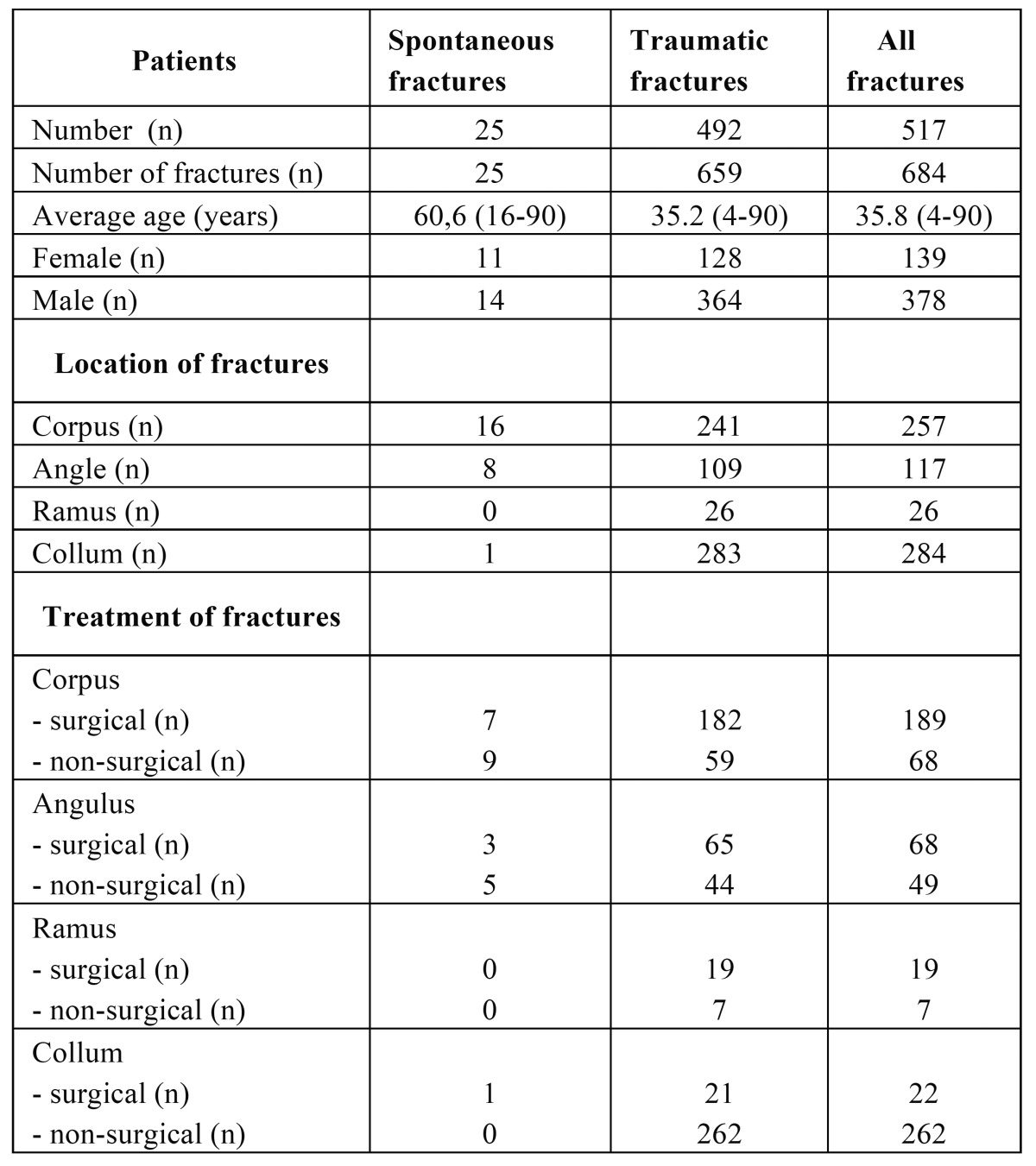
Age, sex, site of fracture, and treatment of spontaneous and traumatic fractures.

**Figure 1:**
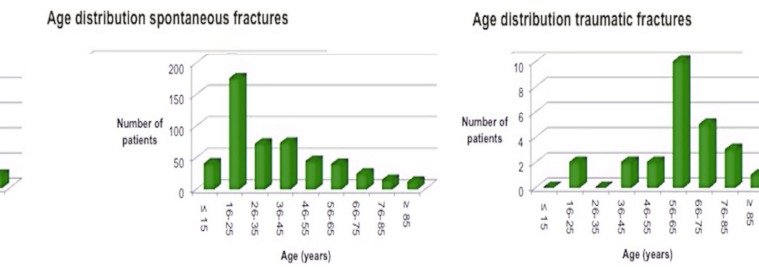
Age related to spontaneous and traumatic fractures of the mandible. Spontaneous fractures occur most frequently in the age group 56-65 years, whereas traumatic fractures are often seen in the age group 16-25 years.

We found 492 patients with 659 mandibular fractures of traumatic origin (accidents, fights or falls); 364 patients (74%) were male and 128 (26%) female, with an average age of 35.2 years (range 4-90) (Fig. 1). The difference in age between the group of spontaneous and traumatic fractures, respectively 60.6 years and 35.2 years in average, was statistically significant (*p*<0,05). There was no statistically significance in relation to gender between spontaneous and traumatic fractures. However, an indication of equal sex-distribution in the group of spontaneous fractures was seen (*p*=0.103), whereas the group of traumatic fractures showed a male dominance.

- Cause of fracture

In patients with spontaneous pathological fractures ( Table 2), eight (32 %) fractures were due to osteoradionecrosis; three (12 %) were the result of direct invasion of a primary malignant tumor; three (12 %) occurred in cystic or other benign lesions; two (8 %) in ARONJ lesions and one (4 %) in a region of the mandible with osteomyelitis.

**Table 2:**
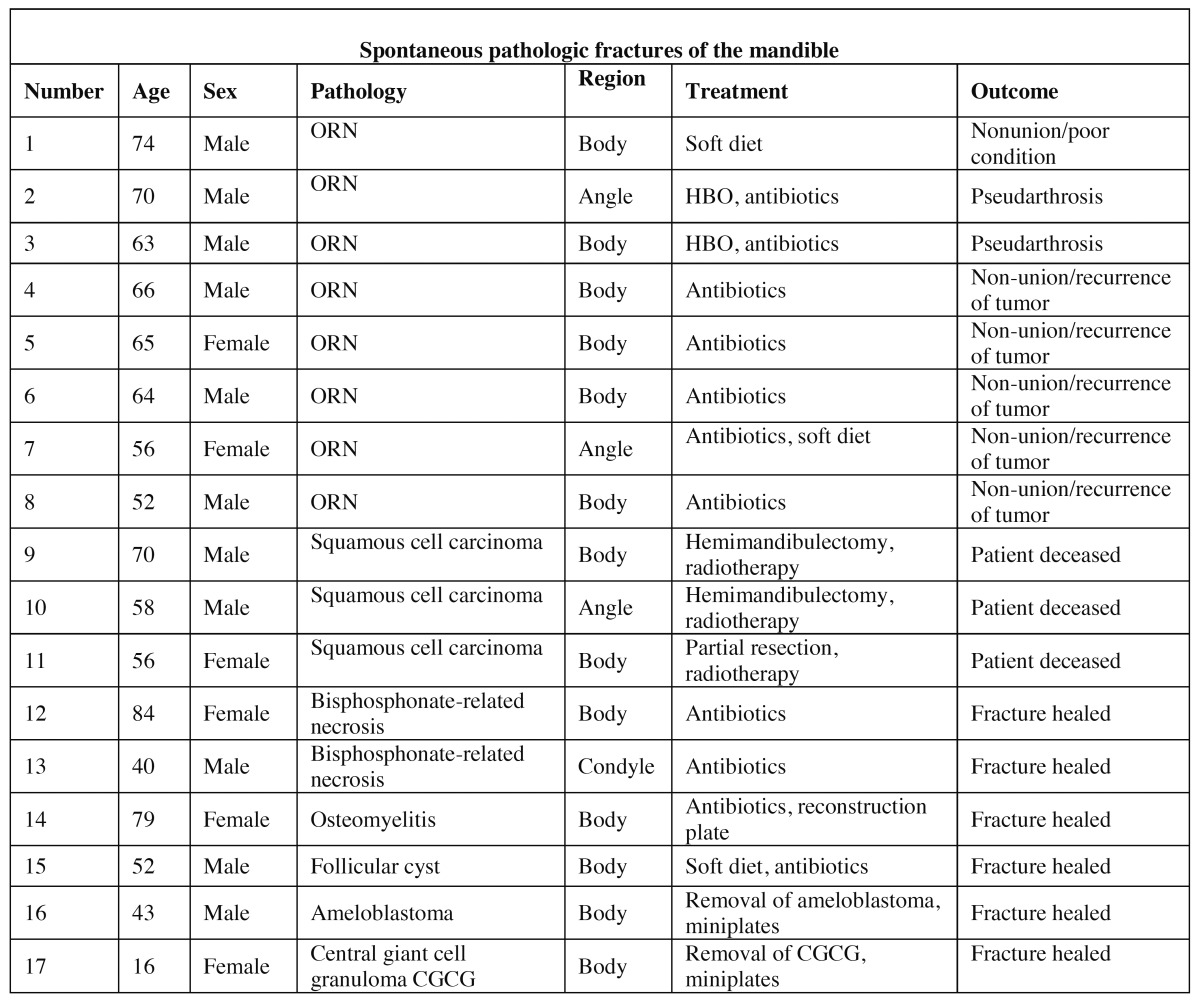
Data concerning 17 spontaneous fractures caused by pathology.

In patients with spontaneous non-pathological fractures ( Table 3), four (16 %) fractures were associated with severe mandibular atrophy of an edentulous mandible. None of these patients had a medical history, which could influence the resorption of bone. Moreover, these fractures were not included unless they had occurred during normal jaw function without any traumatic force applied. Four (16 %) fractures occurred spontaneously following removal of a third molar. None of these patients showed signs of osteomyelitis.

**Table 3:**
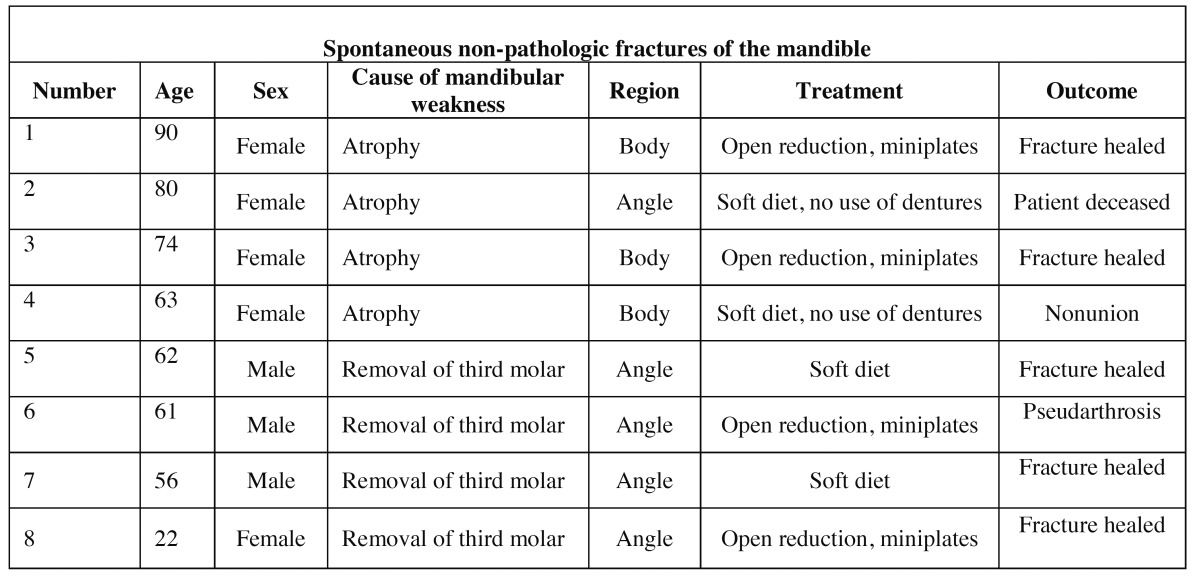
Data concerning 8 spontaneous fractures caused by weakening of the bone.

- Treatment strategy

**Figure 2:**
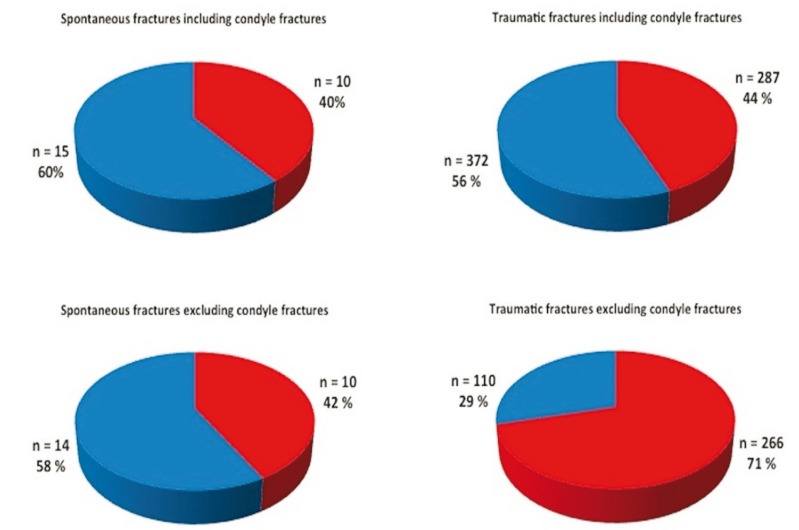
Treatment of spontaneous and traumatic fractures with inclusion and exclusion of condyle fractures. The diagrams show surgical treatment (red) and non-surgical treatment (blue) of spontaneous and traumatic fractures by inclusion and exclusion of condyle fractures. When fractures of the condyle are not included, the number of patients with spontaneous fractures treated with or without surgery is almost unaffected, whereas a marked change in traumatic fractures occurs.

Of the 25 patients with 25 spontaneous fractures, 15 fractures (60 %) were treated non-surgically and 10 fractures were (40 %) surgically treated. Of the 492 patients with 659 traumatic fractures, we found 372 fractures (56%) treated non-surgically with intermaxillary fixation and/or restricted diet; these were typically unilateral high/intracapsular fractures of the mandibular joint or non-displaced fractures of the corpus/angulus. We found 287 mandibular fractures (44%) of traumatic origin treated surgically (Fig. 2). The difference in treatment strategy was not significant.

The majority of the 17 spontaneous pathological fractures were found in an osteoradionecrotic lesion (n=8). In our study, the patients’ general health status or reoccurrence of primary cancer impeded surgical treatment in six patients. Another two patients with osteoradionecrosis had no symptoms after hyperbaric-oxygen treatment and refrained from further treatment.

Three patients with fractures caused by squamous cell carcinoma in the floor of the mouth and invasion of mandibular bone underwent hemi-mandibulectomi or segmental resection. No secondary reconstruction was performed as these patients deceased after adjuvant radiotherapy.

Two patients with ARONJ received a high dose of long-term antibiotic treatment, after which their fractures consolidated with no further treatment required.

One fracture in an osteomyelitic lesion was treated with open reduction and fixation using a reconstruction plate and the patient was given a high-dose of long-term antibiotics.

Fractures caused by ameloblastoma (n=1) and central giant cell granuloma (n=1) were treated with open reduction and fixation with mini plates and mono cortical screws after removal of the pathological lesion. Likewise, a fracture occurring after cystectomy of a follicular cyst was treated with open reduction and fixation with mini plates and mono cortical screws.

Two of four patients with spontaneous non-pathological fractures due to severe mandibular atrophy underwent surgery and the fractures were treated by open reduction and fixation with mini plates and mono cortical screws. The general health status of the remaining two patients did not permit general anesthesia and treatment consisted of soft diet and restrain from use of dentures.

In case of displacement, the spontaneous non-pathological fractures related to third molar removal were treated with mini plates and mono cortical screws.

## Discussion

As controversy exists in the literature regarding the definition of a pathological fracture, one aim of this study was to address the terminology of the field. We suggest the term spontaneous fractures covering “fractures occurring during normal jaw function” with a subdivision of spontaneous fractures into pathological caused by pathological alteration of the bone tissue and non-pathological fractures caused by weakening of the bone without any bone pathology. First, defining a spontaneous fracture as occurring during normal jaw function circumvents speculation of how to define inadequate or minimal trauma. Secondly, we think, that our suggestion of an overall term of spontaneous fracture also known from the orthopedic literature, harmonizes the terminology across the specialties.

Treatment strategy of traumatic fractures in the mandible is well established with the aim to restore function and anatomy, and a surgical approach is often the choice of treatment (16). A patient presenting with a traumatic fracture is typically young and healthy, has normal bone anatomy and physiology with a potential of uneventful healing. On the contrary, patients with spontaneous, particularly pathologic fractures, present a much more complex clinical situation since they may have impaired bone-healing capacity due to pathology of the bone, lack of buttressing, and generally a compromised nutritional status or complex medical issues.

For example, the osteoradionecrotic lesion often occurs within the first years after termination of radiotherapy and before the patients have fully recovered after cancer therapy (7). The malignant disease may have lead to malnutrition and progressive weight loos. The subsequent tumor surgery and/or chemo- and radiotherapy cause pain, taste alteration, xerostomia and difficulty in swallowing resulting in further nutritional decline that contribute to reduced immune function and impaired wound healing (17,18). Furthermore, these patients are often elderly and may have a deprived health because of long-standing tobacco and alcohol abuse. Therefore, treatment planning should also be directed towards the patient´s general health, nutritional status and ruling out recurrent cancer. A fracture in an osteoradionecrotic lesion (Marx stage III) often presents with widespread infection in the bone and surrounding soft tissues, and resection with subsequent reconstruction is the treatment of choice (19). In our study, surgical treatment of these patients never became an option due to primary cancer reoccurrence.

Our findings of spontaneous pathological fractures in primary tumors and metastases (12 %) were in accordance with data from other publications (1,4). When a pathological fracture occurs in a resettable malignant tumour, treatment includes radical surgery with segmental resection of the mandible adjuvant postoperative radiation and/or chemotherapy (1).

The medical treatment of patients receiving high dose anti-resorptives may include steroids and chemotherapy (1); a combination that leads to immunosuppression. There is no consensus regarding management of pathologic mandibular fractures in patients with ARONJ lesions. The published strategies range from conservative treatment to major bone resections with or without internal or external fixation and with or without autogenous reconstruction (8).

A mandibular pathological fracture caused by osteomyelitis is managed stepwise: First, antibiotic therapy directed against the causative organism in a minimum of 6 weeks of intravenous therapy (1). Second, treatment of the fracture depends on the amount of viable bone following sequestrectomy or resection (1,4).

In the case of cysts or benign tumors, the main principles are removal of the lesion and provision of stable fixation with or without bone graft (1-3). In the present study, adequate healing was achieved after open reduction and fixation with mini plates and mono cortical screws without any graft.

In the atrophic mandible, lack of buttressing, impaired vascularity and dense cortical bone compromises healing of a spontaneous fracture, which commonly leads to lack of bony union (9). These fractures often occur in elderly in whom operative risk is significant because of, associated systemic diseases (9). Therefore, the treatment of fractures of atrophic mandibles ranges from conservative management with a soft diet to fixation with reconstruction plates or use of mini plates with extra- or intraoral approach (1,4,9,10).

A large defect may be present in the bone of a patient with a mandibular fracture following removal of a third molar. However, these patients are usually in a good health, have viable bone and a simple fracture pattern, which will lead to uneventful healing unless the bone is infected (1,4).

In our study, we found an approximately equal distribution of men and women with spontaneous fractures, whereas other studies found a clear preponderance of men (1,3,4). Our material is small, with diversity in the cause of fractures, which may explain the difference. Spontaneous pathological fractures caused by osteoradionecrosis and spontaneous non-pathological fractures following removal of third molars occur more frequently in men than women (1,3), while fractures due to atrophy and ARONJ are often seen in women (1,3,4).

In consistency with our findings, spontaneous pathological and non-pathological fractures occur almost entirely in patients past middle age, while the majority of mandible and other facial fractures occur in younger people (1,4).

In comparison to fractures caused by any given trauma, spontaneous fractures are often treated non-operatively, explained by the above-mentioned challenges in handling these fractures. Fifteen of 25 (60 %) spontaneous fractures and 372 of 659 (56 %) of the fractures due to trauma were managed with a non-operative approach. This difference was not statistically significant. However, in our as well as comparative studies (1,3,4), spontaneous fractures of the condyle are infrequent while condyle fractures accounted for approximately half of the traumatic fractures. Although the trend is shifting towards surgical treatment, unilateral high or intra-capsular condyle fractures are often treated non-operatively (20). Thus, if we exclude condyle fractures from both groups, we obtain a more accurate comparison of the treatment approach between spontaneous and traumatic fractures. Then, 14 out of 24 (58 %) spontaneous fractures and 110 out of 376 (29 %) fractures caused by trauma were treated non-operatively (Fig. 2). This difference is statistically significant (*p*<0,05).

## Conclusion

The study showed, that a non-operative approach was more frequent in mandibular spontaneous fractures than in fractures caused by trauma. Moreover, the challenges treating these fractures were discussed.

We addressed the terminological controversies of the field, and have suggested the term spontaneous fractures as “a fracture occurring during normal jaw function” being either pathological or non-pathological.
